# Editorial: Secondary metabolites in grapevine stress response - women in plant science series

**DOI:** 10.3389/fpls.2023.1272668

**Published:** 2023-09-15

**Authors:** Alessandra Ferrandino, Chiara Pagliarani

**Affiliations:** ^1^ Department of Agricultural, Forest and Food Sciences, University of Torino, Grugliasco, Italy; ^2^ Institute for Sustainable Plant Protection, National Research Council (IPSP-CNR), Torino, Italy

**Keywords:** grapevine biotic stress, abiotic stress, vineyard, berries, vegetative organs, plant hormones

The ten papers included in this Research Topic focus on two main subjects, one dealing with the effects of climate change-induced stress on the vine’s secondary metabolism, and one concerning changes in secondary metabolites associated with the grapevine response to pathogens.

Half of the contributions falls in the framework of the first theme and describes either the impact of abiotic factors on the accumulation of secondary metabolites in berries or the application of innovative vineyard management strategies to mitigate the effects of climate alterations on the vine agronomic performance.

A second set of articles reports novel findings on the characterization of molecules involved in grapevine defence process and on the evaluation of natural compounds acting as resistance elicitors, alternative to the application of conventional chemical products.

Schmuleviz et al. evaluated the influence of environmental temperature on the berry metabolic composition of *Vitis vinifera* cv Corvina during postharvest dehydration. The trials were conducted within dedicated dehydration rooms mirroring the environmental conditions typical of two different sites of Northeastern Italy. They dissected the temperature effect from that of the dehydration rate, by excluding the influence of environmental relative humidity. Grape dehydration at higher temperatures (12 °C) promotes the accumulation of oligomeric stilbenes, in parallel with the upregulation of *PAL* and *STS* genes. Conversely, withering the grapes in a cooler environment (8 °C) favors the accumulation of organic acids, flavonols and aromatic compounds.

Variable vineyard microclimatic conditions largely influence berry qualitative traits: Ghiglieno et al. described the effects of strong defoliation on the concentration of some norisoprenoids in Pinot noir and Chardonnay berries, highlighting that high amounts of these compounds are detrimental in sparkling wines, particularly when associated to a drastic reduction of acid concentrations. Asproudi et al. found higher variability among vintages in the accumulation of aroma in berries collected from young Grignolino plants respect to the old ones, whose berries reached a higher concentration of terpenoids. Heat waves with temperatures higher than 30°C, together with water scarcity, vary the berry aromatic profile by increasing the accumulation of benzene derivatives.

Late-pruning is a recent technique proposed to adapt viticulture to climate change: in semi-desert climatic conditions, Perin et al. tested three times of late pruning (1, 2 and 3 weeks after bud-break), highlighting that late pruning decouples the berry ripening dynamics of sugars and phenols, particularly in Syrah respect to Malbec (both grafted onto 110 Richter), resulting in a general increase of polyphenols at harvest. Late pruning in Syrah induced consistent increases in anthocyanins and flavonols, whereas in Malbec significant variation in hydroxycinnamic acid concentration occurred.

Rodas et al. applied a crop-forcing technique, consisting in hedging the growing shoots to seven nodes and removing all lateral leaves and clusters to force the bursting of the primary buds in cv Tempranillo, grown in semi-arid climatic conditions. Although a significant season effect was found for almost all the analyzed polyphenols, a general increase in polyphenol concentration was observed in the wines obtained from forced-vines.

An in-depth analysis of grapevine metabolic compounds acting as resistance trait-associated markers is the subject of the paper by Ciubotaru et al. The objective was to characterize the poorly explored metabolic signature of cultivars with one gene of resistance and with pyramided resistance in comparison with the susceptible variety Teroldego upon *Erysiphe necator* infection. Both timing and intensity of metabolite accumulation control the initiation and the establishment of defense responses, discriminating the resistant individuals from the susceptible ones. Ten metabolites, including pallidol and astringin, were exclusively up-accumulated in the resistant genotypes, suggesting their key biological role in the activation of resistance mechanisms.

*Rugulopteryx okamurae* (Ro) is a species of brown macroalgae belonging to the *Dictyotaceae* family and native to the north-western Pacific, detected at Gibraltar since 2015 and then found on the Andalusian coasts (Zarraonaindia et al.). Its use as a grapevine protection product in viticulture could represent a successful example of circular economy, acquired that some Ro extracts can effectively induce the transcription of defense genes, such as *PR10, PAL, STS48* and *GST1*, and the consequent accumulation of specific secondary metabolites.

The review article by Ferrandino et al. showcases subjects belonging to both the first and the second main themes addressed by this Research Topic, reaffirming how important is to deepen the knowledge on the biological processes that regulate grapevine stress resilience. The review first considers the impact of environmental cues on the berry secondary metabolism, focusing on the molecular and hormonal signalling cascades that control the accumulation of specific groups of quality- and/or defence-associated molecules. Then, the authors analyse recent findings concerning alterations in secondary metabolic compounds that could underlie tolerance/resistance to diseases.

A further and transversal sub-group outlines advances on the genetic regulation of specialized classes of secondary metabolites, namely monoterpenes and sesquiterpenes. The functional plasticity of the numerous terpene synthases and the significant duplication of genes encoding structurally diverse terpene synthases (Bosman et al.) influence product and substrate specificity, impacting on cultivar-specific aroma profiles, a pivotal trait for defining grape quality. However, mono- and sesquiterpenes do play important ecophysiological roles: attraction of pollinators, agents of seed dispersal and herbivores, defense against fungi, promotion of mutualistic rhizobacteria interaction, and their concentration increases upon high light radiation (Bosman and Lashbrooke).

In this subgroup, as well as in all the papers, the paradigmatic nature of this Research Topic is evident: the secondary metabolites that the vine accumulates in its various organs characterize the different genotypes, the result of *millennia* of acclimation. During its evolution, the vine had to live by interacting with biotic and abiotic stresses present in the different areas where it developed and was progressively domesticated. The accumulated secondary metabolites have allowed grapevines to refine strategies of struggle and/or coexistence with the environment and with its microbiome, parasites and/or symbionts, giving to the existing genotypes an optimal level of growth/defense tradeoff. This compromise, the result of at least two *millennia* of attempts, is what we find in the secondary metabolites of the grapes and their derivatives in the wines, produced in different *terroirs*, and what allows the attentive taster to recognize the grape origin of the grapes and their history of adaptation to the environment, cultivation and winemaking.

## Author contributions

AF: Conceptualization, Data curation, Funding acquisition, Writing – review & editing. CP: Conceptualization, Data curation, Funding acquisition, Writing – review & editing.

